# Infection preparedness of community health workers: implications for maternal and neonatal health services in Pakistan

**DOI:** 10.1017/S1463423622000081

**Published:** 2022-05-02

**Authors:** Sara Rizvi Jafree, Amna Khawar, Ain ul Momina, Qaisar Khalid Mahmood

**Affiliations:** 1 Department of Sociology, Forman Christian College University, Lahore, Pakistan; 2 Department of Psychology, Lahore College of Women University, Lahore, Pakistan; 3 Institute of Public Health, King Edward Medical University; 4 Department of Sociology, International Islamic University, Islamabad

**Keywords:** community health workers, coronavirus preparedness, coronavirus responsiveness, employee satisfaction, maternal and neonatal health, Pakistan

## Abstract

**Aim:**

This study aimed to (i) identify community health workers’ (CHWs) perceived satisfaction for maternal and neonatal health services, with respect to (1) socio-demographic characteristics; (2) coronavirus preparedness; (3) coronavirus responsiveness; and (4) employee satisfaction and (ii) investigate the interplay among study variables to identify the role of direct effects and mediation.

**Background:**

Women CHWs are salient providers for maternal and neonatal services at the primary level, especially in conservative regions. Service delivery is a valuable indicator for mother and newborn wellbeing. There is need for empirical evidence to understand how CHWs may be supported in delivering maternal and neonatal health services during pandemics.

**Methods:**

Bivariate regression was used to identify the lower odds for CHWs’ perceived satisfaction for maternal and neonatal health services. In addition, structural equation modeling was used to investigate if coronavirus responsiveness and employee satisfaction as mediating variables influence the relationship between coronavirus preparedness and maternal and neonatal health services. Data were collected telephonically from 350 CHWs. The sample was representative of 35 districts of Punjab, which is the most populated province in the country.

**Findings:**

We found thirty predictors with respect to coronavirus preparedness, coronavirus responsiveness and employee satisfaction which contribute to lower odds of satisfaction for maternal and neonatal health services. We also found that coronavirus preparedness has a direct effect on maternal and neonatal health service satisfaction (*β* = .242, *P* < .001) and an indirect effect on maternal health satisfaction (*β* = .242, *P* < .001) via the mediation of employee satisfaction. We conclude with four critical recommendations to support CHWs in delivering optimal services, comprising of education and training, operational support, public acceptance, and employee support and benefits. The findings are important for the planning of primary health services and governance support for CHWs and poor women clients in Pakistan and other developing countries.

## Introduction

Women community health workers (CHWs) are instrumental in improving maternal and neonatal health indicators in conservative regions like Pakistan and South Asia where the mobility of women is restricted due to cultural and religious interpretations and women are dependent on health services at the doorstep (Gilmore and McAuliffe, 2013). The additional and essential role that CHWs are expected to perform during the coronavirus pandemic is creating awareness and ensuring prevention for infection control in underprivileged and semi-literate communities (Bhaumik *et al*., 2020). Recent scholarship highlights that there is greater vulnerability to mothers and newborns during pandemics in South Asia and other developing countries and also greater risk of still-births (McClure *et al*., 2020).

Doorstep services in the community during pandemics are not just essential for reproductive health services but also for the protection of mother, newborn and entire families through guidance about both infection control and coronavirus symptom management (Webber and Chirangi, 2020). In Pakistan, women CHWs play a critical role in facilitating access to primary healthcare for women, and in addition, they are the only healthcare support for majority of impoverished women in the country (Shaikh and Hatcher, 2005). The Pakistan Ministry of National Health Services Regulation and Coordination launched the National Programme for Family Planning and Primary Health Care, commonly referred to as the Lady Health Workers (LHWs) Program, in 1994. This program has successfully deployed more than 110 000 CHWs across disadvantaged communities of the country (Farooq and Arif, 2014).

After recruitment, CHWs receive 15 months of training and are designated to visit 1,500 women in the community to provide antenatal, natal and postnatal services (Hafeez *et al.*, 2011). They are also responsible for referral to nearby health facilities and provision of health education, including infection control and prevention (Douthwaite and Ward, 2005). However, CHWs in Pakistan are known to face considerable challenges while delivering services, mainly that of low pay and community resistance in accepting services from non-traditional agents (Haq *et al*., 2008; Closser and Jooma, 2013). Another issue is the low-quality training they receive at induction and the drop in their knowledge and skill set due to non-existence of a regular training system (Oxford Policy Management, 2009; Jalal, 2011).

Despite the challenges they face, evidence shows that CHWs have been effective in some indicators for maternal and neonatal health, such as increasing tetanus coverage, immunization, attended deliveries and exclusive breastfeeding (Jalal, 2011). With regard to areas related to infection control, CHWs have also been evidenced to improve women’s awareness and practices in sterilizing drinking water and improving hygiene (Rabbani *et al*., 2016a). In the age of coronavirus, the impact of CHWs services assumes greater significance with regard to educating mothers in the community about coronavirus prevention and management.

After the 18^th^ constitutional amendment in 2011, the subject of health was devolved to provinces. In 2013, the provincial government of Punjab integrated the LHWs program with three other programs: (i) The MNCH (Maternal Neonate Child Health); (ii) Nutrition enhancement; and (iii) The Basic EmONC (Emergency Obstetric Care) program. This new integrated system is called the Integrated Reproductive Maternal Neonatal Child Health (IRMNCH & NP) Programme (Nishtar, 2011). In Punjab, 78% of rural and 30% of the urban population is covered by CHWs across the 36 districts of the province (Oxford Policy Management, 2009). Post the spread of the coronavirus pandemic in the country, there have been guidelines issued by The Primary and Secondary Healthcare Department (Punjab Government, 2020). However, the major limitation is that no formal training has been carried out, and only guidelines for prevention, sanitation and symptom management have been distributed through booklets to CHWs. There has been no investigation about the efficacy and limitations of these guidelines, additional needs for preparedness and response, or the quality of primary healthcare services for maternal and newborn health during the pandemic.

### Study aims

The perceived satisfaction of CHWs in delivering maternal and neonatal health services is an important indicator of mother and newborn wellbeing (Wilford *et al*., 2018). In the absence of sufficient research during the coronavirus pandemic (Singhal, 2020), it is important for independent researchers to help in filling the gaps about how maternal and neonatal health services are influenced by coronavirus preparedness and responsiveness in CHWs. At first step, we aimed to identify the lower odds for CHWs’ perceived satisfaction for maternal and neonatal health services, with respect to four areas: (1) socio-demographic characteristics; (2) coronavirus preparedness; (3) coronavirus responsiveness; and (4) employee satisfaction.

Local literature highlights that CHWs face considerable occupational challenges (Hafeez *et al.*, 2011) and that their service response and employee satisfaction may influence maternal and neonatal health services. Thus, at second step we aimed to investigate the interplay among study variables by performing structural equational modeling (SEM). In this way, the objective of our fifth and last research question was to examine (5) how coronavirus responsiveness and employee satisfaction, as mediating variables, influence the relationship between coronavirus preparedness (independent variable) and maternal and neonatal health services (dependent variable). We believe our study is important not just for healthcare practitioners with weak bargaining power and inadequate governance voice in South Asia and other developing countries (Iacobucci, 2020) but also for the poor women dependent on primary healthcare services in low-income communities (Hick and Biddinger, 2020). Overall, the findings of our study will be useful for the planning of primary health services delivered by women CHWs across South Asia and other developing regions.

## Methods

This study adopts a cross-sectional quantitative design. Ethics approval was taken from the Institutional Review Board, Forman Christian College University. A cover letter was provided to CHWs describing the study and informed consent was taken (Panter and Sterba, 2011). No names were taken from the respondents, and there was no risk to their safety. Respondents were assured that they could withdraw from the study at any point during the telephonic interview. No incentives were offered for participation in this study.

### Sample

The selection criterion for this study was all currently working, government employed, CHWs called LHWs providing outreach services at the doorstep of the community. A total of 44 700 LHWs are deployed in the rural and urban slums of Punjab (Kayani *et al*., 2016). The target sample for this study based on Taros sampling formulae and population of LHWs in Punjab was estimated at 327 (Tepping, 1968).

### Measures

The survey included questions from three standardized tools and consisted of 58 items. There were 6 questions addressing socio-demographic characteristics of respondents (Appendix A).

### Coronavirus preparedness and responsiveness

Coronavirus preparedness and responsiveness was measured using 27 questions from the Zika Outbreak Emergency Preparedness and Response Survey (Rajiah *et al*., 2019). This survey includes items from a checklist developed by The Center for Disease Control and Prevention and World Health Organization, which assesses how prepared healthcare professionals are for a pandemic outbreak. Minor modifications for relevancy to coronavirus were made. A five-point Likert scale was used ranging from ‘strongly disagree’ to ‘strongly agree’. A sample item for measuring coronavirus preparedness was ‘*I know all the information about coronavirus preparedness related to my community needs’*, and a sample item for coronavirus responsiveness was ‘*I can manage the common symptoms and reactions of coronavirus’*.

### Employee satisfaction

Employee satisfaction was measured using 13 questions from the Community Health Worker Employer Survey (Chaidez *et al*., 2018). A five-point Likert scale was used ranging from ‘strongly disagree’ to ‘strongly agree’. This measure took into consideration employee support from coworkers and supervisors, and satisfaction with regard to workload, pay and contractual benefits. Items included statements like ‘*My supervisor/team leader treats me with respect’* and ‘*My workload is reasonable’*.

### CHW satisfaction with maternal and neonatal health services delivered

CHW satisfaction with maternal and neonatal health services delivered was measured using the Self-reported Performance of maternal and child health Workers-Nepal scale (Chhetry *et al*., 2005). The measure included 12 items related to satisfaction with antenatal care, postnatal care, emergency care, birthing care and newborn care. A five-point Likert scale was used ranging from ‘strongly disagree’ to ‘strongly agree’. The measure included items like ‘*I am satisfied with delivery of services for prior referrals for birth care’* and ‘*I am satisfied with delivery of services for birth complications managed and/or referred’*.

### Data collection

We requested a list of mobile contact numbers of CHWs from the IRMNCH & NP, Punjab, and were able to gain access to a list of 1,000 numbers. The authors of the study recruited and trained 12 women research assistants for the data collection, during a two-week period through zoom video sessions. The research assistants were University students of Psychology experienced in data collection. The data were collected during the months of May 2020 to June 2020, using telephonic survey method, to observe physical distancing and safety during the coronavirus pandemic. Initially, we text-messaged the entire contact list informing CHWs of the research objectives, and seeking their permission for participation in the study (Delice and Practice, 2010). We followed up with one text message when we did not receive a reply. A total of 373 CHWs replied and gave consent to be interviewed, and we were finally able to collect complete data from 350 women, making the final response rate for this study 35%. The responding CHWs belonged to 35 of the 36 districts of the province, divided into North and South Punjab (Appendix B).

### Data analysis

We used SPSS 21.0 for analysis of descriptive statistics and bivariate regression. The independent variables for the study include ‘coronavirus preparedness’, ‘coronavirus responsiveness’ and ‘employee satisfaction’, and the dependent variable for the study was ‘satisfaction with maternal and neonatal health services’. Reliability statistics for the scales in the study show good reliability above values of 0.71 (Terwee *et al*., 2007). The overall internal consistency ranged from 0.764 to 0.878 (Table 1). At first step, descriptive statistics were derived. Then, study variables were compounded to assess association between variables, and linear regression was calculated in order to ascertain the direction of relationship.

**Table 1.** Reliability statistics for study domains

Variable	Cronbach’s Alpha	Items
Coronavirus preparedness	0.840	16
Coronavirus responsiveness	0.878	11
Employee satisfaction	0.764	13
Maternal and neonatal health satisfaction	0.810	11

Next, we calculated bivariate odds regression, by recoding study variables into binary categories. We created dummy variables with ‘0’ representing low odds of satisfaction and ‘1’ representing higher odds of satisfaction. Significance of the main effects was estimated by computing the confidence levels. *P*-values of less than 0.05 were considered significant for this study. For adjusted odds ratios (AOR), age and serving years, as continuous variables, were held constant. In the third phase of our analysis, the complex relationships among variables, as well as their determinants, were calculated along with the parameter estimates of the structural model using a path diagram. The authors used AMOS software (version 17.0) for SEM analysis (Byrne, 2001) and entered coronavirus preparedness as the independent variable and satisfaction for maternal and neonatal health services as the dependent variable. Coronavirus responsiveness and employee satisfaction were entered as mediating variables. We opted for maximum likelihood estimation method and performed the bootstrapping on 2000 samples with 90 percent confidence intervals.

## Results

### Descriptive statistics

From a sample of 350 women, majority at 71.1% belonged to North Punjab and were between the ages of 40–59 years. Most CHWs have a secondary (74.0%) or intermediate level degrees (17.4%), and majority had been serving between 1–9 years. Most CHWs are married at 84.3% and have less than 3 children, at 64.6% (Table 2).

**Table 2.** Socio-demographic characteristics of sample, *n* = 350

Variable	Frequency (%)
District	
North Punjab	249 (71.1%)
South Punjab	101 (28.9%)
Age	
17–39	100 (28.6%)
40–59	250 (71.4%)
Degree	
Secondary (grade 10)	259 (74.0%)
Intermediate (grade 12)	61 (17.4%)
Bachelors-Masters	30 (08.6)
Serving years	
01–19	254 (72.6%)
20–38	96 (27.4%)
Marital status	
Married	295 (84.3%)
Unmarried	55 (15.7%)
Number of children	
Less than three	226 (64.6%)
More than three	124 (35.4%)

### Correlation and linear results

Table 3 presents the mean and standard deviations of study variables and the Pearson correlation results. All study variables have significant Pearson’s correlation associations, above cutoff values of 0.300 (Schober *et al*., 2018), ranging from 0.304 to 0.768. Linear regression results showed that maternal and neonatal health satisfaction is positively associated with independent variables of (i) coronavirus preparedness (*R*^2^ = 0. 128); (ii) coronavirus responsiveness (*R*^2^ = 0.122); and (iii) employee satisfaction (*R*^2^ = 0. 115).

**Table 3.** Pearson’s correlation results for study domain variables

	Mean	SD	CP	CR	ES	M&NHS
Coronavirus preparedness (CP)	67.48	6.881	1			
Coronavirus responsiveness (CR)	45.09	5.497	.717**	1		
Employee satisfaction (ES)	53.08	7.690	.578**	.689**	1	
Maternal and neonatal health satisfaction (M&NHS)	27.12	2.616	.358**	.349**	.340**	1

** *P* value < 0.005.

### Socio-demographic regression results

In Table 4, we present the results for higher odds of CHWs’ satisfaction for maternal health services and neonatal health services with respect to socio-demographic characteristics. We found no significant associations.

**Table 4.** Bivariate regression results for lower odds of CHWs’ satisfaction for maternal health services and neonatal care services, with respect to socio-demographic characteristics

Variable	Low odds of satisfaction for maternal & neonatal health service	
	OR (CI) *P*-value	AOR* (CI) *P*-value
District		
North Punjab South Punjab	1.41 (0.82–2.43), 0.212	1.39 (0.81–.2.41), 0.230
Degree		
Secondary-Intermediate Bachelors-Masters	1.12 (0.46–2.73), 0.790	1.09 (0.44–2.66), 0.848
Marital status		
Married Unmarried	2.05 (0.88–4.75), 0.092	2.18 (0.93–5.08), 0.071
Number of children		
Less than three More than three	1.28 (0.75–2.17), 0.351	1.33 (0.77–2.29), 0.297

*For adjusted odds ratio calculation, age and service years, as continuous variables, have been kept constant.

### Coronavirus preparedness regression results

With regard to results for adjusted odds ratio results for coronavirus preparedness (Table 5), we find that CHWs have lower odds of satisfaction with maternal and neonatal health services when they (i) do not have all the information about coronavirus preparedness related to community needs (AOR: 8.20; 95% CI 3.09–13.74); (ii) do not know how to advise about distancing to minimize risks of community exposure (AOR: 5.33; 95% CI 1.16–9.56); (iii) do not have access to journal articles related to coronavirus preparedness (AOR: 5.31; 95% CI 2.84–9.90); (iv) do not know about decontamination procedures (AOR: 5.20; 95% CI 1.10–9.51); (v) are not familiar with the local emergency response for coronavirus (AOR: 3.70; 95% CI 1.82–7.53); (vi) are not prepared for the management of coronavirus (AOR: 3.68; 95% CI 1.97–6.87); (vii) do not have sufficient support from local officials in an emergency (AOR: 3.38; 95% CI 1.54–7.40); (viii) are not considered key leaders in the community in coronavirus outbreak (AOR: 2.98; 95% CI 1.65–5.39); (ix) do not have awareness of the programs about CP and management offered by the government (AOR: 2.69; 95% CI 1.47–4.93); (x) do not know who to contact from chain of command in disaster situations (AOR: 2.19; 95% CI 1.13–4.21); and (xi) have not participated in emergency planning for coronavirus situations (AOR: 1.78; 95% CI 1.03–3.10).

**Table 5.** Bivariate regression results for lower odds of CHWs’ satisfaction for maternal health services and neonatal care services, with respect to coronavirus preparedness (CP)

Variable	Low odds of satisfaction for maternal & neonatal health service	
	OR (CI) *P*-value	AOR* (CI) *P*-value
Have all the information about CP related to my community needs		
No Yes	8.02 (3.07–13.95), 0.000	8.20 (3.09–13.74), 0.000
Aware of the obstacles in CP related to community		
No Yes	1.09 (0.35–3.38), 0.873	1.04 (0.33–3.25), 0.937
Aware of educational classes on CP that relate to my community		
No Yes	1.80 (0.93–3.46), 0.078	1.84 (0.95–3.55), 0.069
Aware of the programs about CP management by the government		
No Yes	2.59 (1.43–4.70), 0.002	2.69 (1.47–4.93), 0.001
Have access to journal articles related to CP		
No Yes	5.38 (2.89–8.02), 0.000	5.31 (2.84–9.90), 0.000
Receive sufficient support from local officials in emergency		
No Yes	3.39 (1.55–6.42), 0.002	3.38 (1.54–7.40), 0.002
Have contact of chain of command in disaster situations		
No Yes	2.14 (1.12-4.12), 0.021	2.19 (1.13-4.21), 0.019
Have been participating in educational activities dealing with CP		
No Yes	1.68 (0.84–3.34), 0.137	1.71 (0.86–3.42), 0.123
Have participated in emergency planning for coronavirus		
No Yes	1.75 (1.01–3.02), 0.044	1.78 (1.03–3.10), 0.040
Can take relevant exposure history before home visit		
No Yes	1.81 (0.75–4.37), 0.187	1.82 (0.75–4.43), 0.181
Aware of how to use personal protective equipment		
No Yes	1.88 (0.55–5.42), 0.314	1.74 (0.51–6.03), 0.378
I know how to execute decontamination procedures		
No Yes	5.10 (1.11–9.34), 0.035	5.20 (1.10–9.51), 0.037
I know how to advise about distancing to minimize exposure		
No Yes	5.11 (1.12–9.35), 0.036	5.33 (1.16–9.56), 0.032
I am familiar with the local emergency response system		
No Yes	3.54 (1.76–7.14), 0.000	3.70 (1.82–7.53), 0.000
I am considered a key leader in my community		
No Yes	2.93 (1.63–5.27), 0.000	2.98 (1.65–5.39), 0.000
I consider myself prepared for the management of coronavirus		
No Yes	3.70 (2.01–6.83), 0.000	3.68 (1.97–6.87), 0.000

*For adjusted odds ratio calculation, age and service years, as continuous variables, have been kept constant.

### Coronavirus responsiveness regression results

Adjusted odds ratio results for coronavirus responsiveness (Table 6) show that CHWs have lower odds of satisfaction with maternal and neonatal health services when they (i) are not familiar with the scope of their role in coronavirus (AOR: 5.60; 95% CI 2.14–9.65); (ii) are not confident in their abilities in coronavirus as a member of a healthcare team (AOR: 5.16; 95% CI 2.51–7.62); (iii) are not confident in their abilities as a direct care provider or first responder in coronavirus (AOR: 2.85; 95% CI 1.49–5.45); (iv) cannot manage the common symptoms and reactions of coronavirus (AOR: 2.51; 95% CI 1.45–4.34); (v) are not confident implementing plans for social distancing, infection control, hygiene literacy, & similar functions (AOR: 2.45; 95% CI 1.10–5.47); (vi) cannot identify possible indicators of mass exposure evidenced by a clustering of patients with similar symptoms (AOR: 2.79; 95% CI 1.39–5.57); (vii) as healthcare practitioners, do not feel confident as a manager or coordinator of a community exposed to coronavirus (AOR: 3.14; 95% CI 1.44–6.83); (viii) are not provided opportunities to participate in peer evaluation of skills and governance planning on coronavirus (AOR: 3.50; 95% CI 1.49–8.23); and (ix) are not accepted as a legitimate authority for coronavirus awareness/prevention in the community (AOR: 3.43; 95% CI 1.91–6.15).

**Table 6.** Bivariate regression results for lower odds of CHWs’ satisfaction for maternal health services and neonatal care services, with respect to coronavirus responsiveness

Variable	Low odds of satisfaction for maternal & neonatal health service	
	OR (CI) *P*-value	AOR* (CI) *P*-value
I am familiar with the scope of my role as a healthcare provider		
No Yes	5.73 (2.21–9.84), 0.000	5.60 (2.14–9.65), 0.000
I am confident in my abilities as a member of a healthcare team		
No Yes	5.14 (2.51–7.52), 0.000	5.16 (2.51–7.62), 0.000
I am confident as a direct care provider or first responder in coronavirus		
No Yes	2.86 (1.51–5.39), 0.001	2.85 (1.49–5.45), 0.001
I can care for coronavirus patients independently without any supervision		
No Yes	1.59 (0.99–2.69), 0.081	1.60 (0.94–2.72), 0.080
I can manage the common symptoms and reactions of coronavirus		
No Yes	2.47 (1.44–4.24), 0.001	2.51 (1.45–4.34), 0.001
I feel confident implementing plans for social distancing, infection control, hygiene literacy, & similar functions		
No Yes	2.31 (1.04–5.11), 0.038	2.45 (1.10–5.47), 0.028
I can identify possible indicators of mass exposure evidenced by a clustering of patients with similar symptoms		
No Yes	2.71 (1.36–5.39), 0.005	2.79 (1.39–5.57), 0.004
As a HCP, I would feel confident as a manager or coordinator of a community exposed to coronavirus		
No Yes	2.99 (1.39–6.43), 0.005	3.14 (1.44–6.83), 0.004
I am provided opportunities to participate in peer evaluation of skills and governance planning on coronavirus		
No Yes	3.46 (1.48–8.09), 0.004	3.50 (1.49–8.23), 0.004
I am familiar with how to perform focused health assessment for coronavirus		
No Yes	1.89 (0.96–3.73), 0.063	1.88 (0.95–3.72), 0.068
I am accepted as a legitimate authority for coronavirus awareness/prevention in the community		
No Yes	3.40 (1.90–6.08), 0.000	3.43 (1.91–6.15), 0.000

*For adjusted odds ratio calculation, age and service years, as continuous variables, have been kept constant.

### Employee satisfaction regression results

Adjusted odds ratio results for employee satisfaction (Table 7) show that CHWs have lower odds of satisfaction with maternal and neonatal health services when (i) working relationship with coworkers is not good (AOR: 11.59; 95% CI 3.55–18.78); (ii) supervisors do not treat them with respect (AOR: 7.94; 95% CI 2.30–14.38); (iii) they do not believe their profession is a good place to work (AOR: 5.97; 95% CI 2.17–8.39); (iv) communication between senior leadership and employees is not good (AOR: 5.00; 95% CI 2.39–8.48); (v) supervisors do not work well with employees of different backgrounds (AOR: 4.42; 95% CI 1.42–8.72); (vi) they are not provided opportunities to demonstrate their leadership by supervisor (AOR: 3.63; 95% CI 1.82–7.21); (vii) supervisors do not support employee development (AOR: 3.49; 95% CI 1.66–7.33); (viii) supervisor do not support balance in work and family issues (AOR: 2.96; 95% CI 1.69–5.19); (ix) job security and contract is not satisfactory (AOR: 2.49; 95% CI 1.46–4.26); and (x) they cannot learn from coworkers as they do their work (AOR: 1.99; 95% CI 1.01–3.94).

**Table 7.** Bivariate regression results for lower odds of CHWs’ satisfaction for maternal health services and neonatal care services, with respect to employee satisfaction

Variable	Low odds of satisfaction for maternal & neonatal health service	
	OR (CI) *P*-value	AOR* (CI) *P*-value
My profession is a good area to work in		
No Yes	5.89 (2.16–8.06), 0.001	5.97 (2.17–8.39), 0.001
Coworkers & I have a good working relationship		
No Yes	11.64 (3.59–18.75), 0.000	11.59 (3.55–18.78), 0.000
Supervisor treats me with respect		
No Yes	8.09 (2.36–14.66), 0.001	7.94 (2.30–14.38), 0.001
Supervisor provides me with opportunities to demonstrate my leadership		
No Yes	3.63 (1.82–7.21), 0.000	3.63 (1.82–7.21), 0.000
Workload is reasonable		
No Yes	1.20 (0.72–2.01), 0.468	1.20 (0.72–2.01), 0.480
Supervisor supports my need to balance work and family issues		
No Yes	2.81 (1.63–4.86), 0.000	2.96 (1.69–5.19), 0.000
Employees learn from one another as they do their work		
No Yes	1.96 (0.99–3.87), 0.050	1.99 (1.01–3.94), 0.047
Supervisors in my work unit support employee development		
No Yes	3.36 (1.61–7.01), 0.001	3.49 (1.66–7.33), 0.001
Supervisors work well with employees of different backgrounds		
No Yes	4.61 (1.50–8.17), 0.008	4.42 (1.42–8.72), 0.010
Communication between senior leadership and employees is good		
No Yes	5.11 (2.45–8.62), 0.000	5.00 (2.39–8.43), 0.000
Creativity and innovation are rewarded		
No Yes	1.02 (0.58–1.78), 0.935	1.01 (0.57–1.79), 0.958
Pay and employment benefits are reasonable		
No Yes	1.09 (0.65–1.81), 0.738	1.10 (0.66–1.84), 0.699
Job security and contract is reasonable		
No Yes	2.47 (1.45–4.20), 0.001	2.49 (1.46–4.26), 0.001

*For adjusted odds ratio calculation, age and service years, as continuous variables, have been kept constant.

### Structural equation model

Our SEM results for model fit, presented in Table 8, show that all fit indices are within the acceptable limit: [GFI = .998; AGFI = .977; CFI = .999; TLI = .993; RMSEA = .042].

**Table 8.** Model fit indices for SEM

Model fit indices	Value	Suggested cutoff
*χ*^2^ (df)	1.260(1)	Non-significant
Goodness of Fit (GFI)	.998	> .95
Adjusted Goodness of Fit (AGFI)	.977	> .90
Comparative fit index (CFI)	.999	> .90
Tucker–Lewis index (TLI)	.993	> .90
Root mean square error of approximation (RMSEA)	.042	< .08

**Figure 1.**
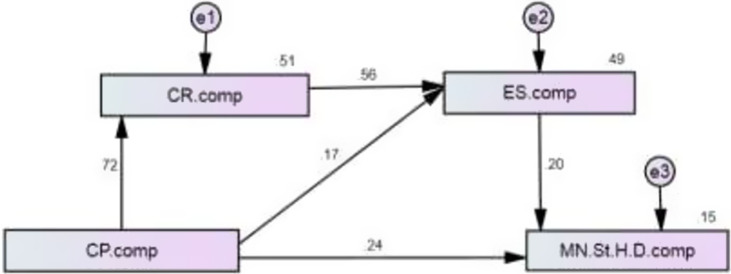
The structural path model for study variables.

Results presented in Table 9 show that coronavirus preparedness has a direct effect on maternal and neonatal health service satisfaction (*β* = .242, *P* < .001) and an indirect effect on maternal health satisfaction (*β* = .242, *P* < .001) via the mediation of employee satisfaction. Coronavirus responsiveness does not have any direct effect on maternal and neonatal health service satisfaction, whereas coronavirus responsiveness has an indirect effect on maternal and neonatal health service satisfaction (*β* = .113, *P* < .001) via the mediation of employee satisfaction. Coronavirus preparedness has a direct effect (*β* = .173, *P* < .001) and also an indirect effect (*β* = .405, *P* < .001) on employee satisfaction via the mediation of coronavirus responsiveness. SEM results are summarized in Figure 1.

**Table 9.** Direct and indirect effects of independent and mediating variables on maternal and neonatal health service satisfaction (M&NHSS)

Model	Direct effects (*β*)	Indirect effects (*β*)	Total effects (*β*)
CR preparedness CR responsiveness	.717***		.717***
CR preparedness employee satisfaction	.173***	.405***	.578***
CR preparedness M&NHSS	.242***	.116***	.358***
CR responsiveness employee satisfaction	.564***		.564***
CR responsiveness M&NHSS		.113***	.113***
Employee satisfaction M M&NHSS	.200***	.	.200***

***P values are significant at < .001.

## Discussion

Our study variables show linear association, providing evidence that when coronavirus preparedness, coronavirus responsiveness and employee satisfaction are high, CHWs perceive their delivery of maternal and neonatal services to be better. Bivariate regression results addressing our four research questions imply considerable policy improvement is needed for CHWs with regard to preparedness and response for infection control and general employee support. Our first research question examined a relationship between socio-demographic variables and lower odds of satisfaction with maternal and neonatal services. The results of the data analysis revealed no significant associations.

The second research question tested the relationship between coronavirus preparedness and lower odds of satisfaction with maternal and neonatal services. We found that CHWs have lower odds of satisfaction when they lack information and education and training programs by the government. Specifically, CHWs have lower satisfaction when they are not trained about how to guide mothers about physical distancing, decontamination and disinfection procedures. There is also lower satisfaction when there is inadequate support from local officials and lack of information about local emergency response and who to contact during emergencies. Other research confirms that maternal health indicators show improvement when the state invests in the training and skill development of primary healthcare workers (Scott *et al.*, 2018). Coordination and collaboration with cross-sector partners in the community is vital for emergency response and effective service delivery (Ransom *et al*., 2008). Additionally, information sharing about local health teams and effective communication between health teams is needed for optimal delivery of services by CHWs. We also found that lower probability of satisfaction is associated with CHWs’ lack of acceptance as key leaders by the community. Local research suggests that CHWs face considerable resistance in certain communities due to patriarchal and traditional forces which prefer local healers (Jafree *et al*., 2020).

The third research question examined the relationship between coronavirus responsiveness and lower odds of satisfaction with maternal and neonatal services. Findings revealed that CHWs have lower odds of satisfaction when they are not confident about their exact role and abilities to identify patients and manage coronavirus in the community. Other research confirms that CHWs need formal training about their roles and responsibilities for coronavirus management (Ajisegiri *et al*., 2020). We also found that lower probabilities for satisfaction were linked with specific problems related to management of symptoms, implementing social distancing, infection spread and hygiene literacy. Unless CHWs have training for coronavirus symptom management and infection control, there is greater risk of disease spread in disadvantaged communities (Perry *et al.*, 2014). We also found that the odds of satisfaction were low when CHWs are not provided opportunities to participate in peer evaluation of skills and governance planning for coronavirus. Other scholarship highlights that when CHWs participate in peer evaluation and governance, there is improved service quality for maternal and child health in the primary health sector (Kaplan *et al.*, 2013).

The fourth research question examined the relationship between employee satisfaction and lower odds of satisfaction with maternal and neonatal services. Results reveal that CHWs have lower odds of satisfaction when they do not have a good working relationship with their coworkers and are unable to learn from each other. Prior research has reported that coworkers can assist in supporting CHWs in dealing suitably with local issues and improving services (Sharma *et al.*, 2014). Satisfaction is also low when supervisors are disrespectful, communication is inadequate, skill development and leadership is not supported, and work-family balance is not reinforced. Other scholarship confirms that supervisor support is essential for CHW service quality, employee outcomes and stability in family and work equation (Jaskiewicz and Tulenko, 2012). Lastly, we found that the odds of satisfaction are low when there is job insecurity and the employment contract is inadequate. Local research shows that there is need for CHWs contracts to be improved with regard to matching income with inflation and improving their career path and professional advancement (Haq *et al.*, 2008).

With regard to our fifth research question, examining if coronavirus responsiveness and employee satisfaction as mediating variables influence the relationship between coronavirus preparedness and maternal and neonatal health service, we are able to conclude two important things. First, coronavirus preparedness has a significant effect on maternal and neonatal health services, and this relationship is mediated significantly by employee satisfaction. Coronavirus preparedness also has a direct effect and indirect effect on employee satisfaction via the mediation of coronavirus responsiveness. We may derive that even when CHWs are prepared for infection control and management, their response may not be optimal, unless they have organizational, supervisor and coworker support (Jaskiewicz and Tulenko, 2012; Rabbani *et al*., 2016b; Cometto *et al.*, 2018). Second, we found that coronavirus responsiveness has an indirect effect on maternal and neonatal health services via the mediation of employee satisfaction. The results confirm that employee satisfaction is a key factor in securing delivery services during emergency and crisis situations in the community, including pandemics (Zhang *et al*., 2016).

### Limitations of study

The limitations of this research include the cross-sectional design and the inability to sample more provinces. However, Punjab comprises 60% of the Pakistani population and has greater CHW deployment compared to other provinces in the country, thus lending strength to our study in terms of representation (Oxford Policy Management, 2009). There is also the limitation of this being a perception-based study and the biases inherent in healthcare provider response, with respect to delivery of services and employer support. For future studies, we recommend interviews with CHW supervisors and women availing services from CHWs. However, overall the findings of this research have value in providing recommendations and impetus for improving: (i) primary-level maternal and neonatal healthcare services for women of Pakistan; (ii) service quality, training and employee support of CHWs; and (iii) coronavirus preparedness and response protocols for CHWs.

Though this research is relevant for the coronavirus pandemic, it also holds value as a contribution for research-based recommendations for future pandemics and infection management in community settings. In addition, the findings have relevancy for other developing countries planning a CHW program to support disadvantaged women with primary health services. Stemming from the findings of this study, a randomized controlled trial for digital healthcare services for women in communities is being conducted by the first author of this study. Future researchers might want to plan mixed methods research and case studies to better recommend policy improvement for the primary health sector in developing regions.

## Concluding recommendations

CHWs can play a critical role in controlling infection and also protecting mothers and newborn during pandemics. Pakistan is lucky to have an existing CHW program across the country, which is managed by The Ministry of National Health Services Regulation and Coordination and the provincial health bodies responsible for community health services. The findings of our study enable us to inform about the needs of CHWs in delivering optimal services for maternal and neonatal health during pandemics. Our recommendations are beneficial for South Asia and other developing regions planning community health services for maternal and neonatal health for disadvantaged women during pandemics. We conclude with four key areas for support to improve maternal and neonatal health services by CHWs during times of pandemics, described below and summarized in Table 10:

1. Education and Training – it is critically needed to improve skill sets for management of coronavirus in the community, identification of mass exposure and development of confidence levels in CHWs as pandemic coordinators. There is also need to provide access to understandable academic material and recent scholarship related to pandemic management and infection material in CHWs who are mostly secondary school graduates and are not highly educated;
2. Operational Support – it is needed to provide clear guidelines for roles and responsibilities during pandemics and for the introduction of routine practice for pandemic management. We also recommend the formation of and regular participation in committees for emergency response and infection control and regular meetings with local officials and CHW teams for improved coordination and pandemic response;
3. Public Acceptance – it is essential that there is community acceptance of CHWs as legitimate authorities and key leaders for infection control for the effectiveness of service delivery. This is possible through social media and community awareness drives by established community notables like religious leaders, elected political leaders and older and trusted male populations within the local districts; and
4. Employee Support and Benefits – we recommend consistent accountability measures of supervisors to prevent disrespect, bullying and discrimination. There is also need for increased communication and team-building initiatives with coworkers in the primary healthcare sector, such as the medical officer in charge, lady health visitor, vaccinators, community midwives, traditional birth attendants, medical technicians and dispensers, district health officer and lady health supervisor. Opportunities for employee development and leadership are also needed, along with support for work-family balance in order to make service delivery more optimal. Finally, there is need for reforms with regard to job stability and career progression.

**Table 10.** Summary recommendations to help improve maternal and neonatal health services of CHWs during pandemics

Education and training
Improve skill set for: - direct care or first response in coronavirus, - management of the common signs and symptoms of coronavirus, - decontamination procedures and - how to provide advice about social distancing to minimize risks of community exposure - information related to specific community needs
Develop proficiencies for identification of mass exposure
Improve confidence as a manager or coordinator of a community exposed to coronavirus
Increasing access to easy to understand educational material related to management of coronavirus management and infection control
Operational support
Provision of role descriptions with sufficient detail about the scope of role during pandemic
Routine practice for implementing plans for pandemic management, including social distancing, infection control, hygiene literacy, & similar functions
Formation and regular participation in: - An advisory committee for an emergency response system for pandemics - A committee for governance planning on coronavirus/infection control - Government programs about coronavirus management
Regular meetings and coordination for: - Peer evaluation of skills and challenges - Identification of community/local officials’ support system and chain of command in an emergency
Public acceptance
Improve community acceptance of CHWs as legitimate authority for coronavirus awareness and prevention
List and advertise CHWs as key leaders in the community for infection control and management
Employee support & benefits
Accountability of CHW supervisor treatment, discrimination, and bullying
Superior-subordinate meeting forums for improved communication
Team building exercises with coworkers, offering opportunities to learn from each other
Introducing opportunities for CHW development and leadership
Support for work-family balance
Job security and introduce a career progression framework for them

## Supplementary material

For supplementary material accompanying this paper visit http://doi.org/10.1017/S1463423622000081. click here to view supplementary material
