# Supplementary material for: Infection preparedness of community health workers: implications for maternal and neonatal health services in Pakistan
Source: Prim Health Care Res Dev. 2022 May 2;23:e27. doi: 10.1017/S1463423622000081 (PMC9112673; doi:10.1017/S1463423622000081)
Supplement: Supplementary file 1 [file phcsup.zip › S1463423622000081sup001.docx]

**Community Health Workers Satisfaction with Service Quality for Maternal and Child Health in the Age of Corona**

# Informed Cover letter and Consent for Survey

Sign or Thumb Impression for Written Consent/Audio recording or electronic Initials

**Thank you** for your valuable time!

Your name is not required and all research analysis will be undertaken with confidentiality and complete anonymity.

You are free to exit the assisted survey data collection at any time during the proceedings.

The questionnaire has been designed to collect information about the challenges you are facing in in delivering services to women in the community during coronavirus pandemic.

The findings from this research will help identify your needs, plan better employment support for you, and improve service delivery standards for your women clients.

Your honest and reliable answers will be appreciated.

In the event that you feel disturbed or upset after answering questions or recalling memories related to lived experiences, you may call or text the researcher for free consultation services from trained psychologists.

Thank you for your time and participation

Dr. Sara Rizvi Jafree, Assistant Professor, Forman Christian College, University

E-mail: [sararizvijafree@gmail.com](mailto:sararizvijafree@gmail.com);

Cell: 0300 400 5740

**Survey**

The questionnaire will be read out telephonically or collected through online methods by the dt collector

Province/City: ____________________________

LHW District/ Community Code: ___________________________

Other Demographic/ Identifier____________

|  | | | | | | ***Coding*** |
| --- | --- | --- | --- | --- | --- | --- |
| *Socio-demographics:* | | | | | |  |
| Q1.Age? | | | | | |  |
| Q2. Last earned degree? | | | | | |  |
| Q3. Years since serving profession? | | | | | |  |
| Q4.Current Marital Status (single/ separated/ widow/ divorced)- | | | | | |  |
| Q5.How many children do you have? | | | | | |  |
| Q6. Religion? | | | | | |  |
| *Please answer with reference to the last* ***two weeks:*** | | | | | |  |
|  | **Strongly disagree** | **Disagree** | **Neutral** | **Agree** | **Strongly Agree** |  |
| **Zika Outbreak Emergency Preparedness and Response Survey** | | | | | |  |
| *Preparedness* | | | | | |  |
| 1. I know all the information about coronavirus preparedness related to my community needs. |  |  |  |  |  |  |
| 2. I am aware of the obstacles in coronavirus preparedness related to my community. |  |  |  |  |  |  |
| 3. I am aware of educational classes on coronavirus preparedness that relate specifically to my  community situation. |  |  |  |  |  |  |
| 4. I am aware of the programs about coronavirus preparedness and management that are offered  by the government. |  |  |  |  |  |  |
| 5. I read journal articles related to coronavirus virus preparedness. |  |  |  |  |  |  |
| 6. In case of an emergency situation, there is sufficient support from local officials in this region. |  |  |  |  |  |  |
| 7. I know who to contact (chain of command) in disaster situations in my community |  |  |  |  |  |  |
| 8. I participate in educational activities dealing  with coronavirus preparedness on a (continuing education classes, seminars, or conferences). |  |  |  |  |  |  |
| 9. I have participated in emergency plan drafting and emergency planning for coronavirus situations in my community |  |  |  |  |  |  |
| 10. Before home visit, a relevant exposure history should be taken (including exposure criteria of whether the patient has resided in or travelled to a country/ another region) |  |  |  |  |  |  |
| 11. I know how to use personal protective equipment. |  |  |  |  |  |  |
| 12. I know how to execute decontamination procedures. |  |  |  |  |  |  |
| 13. I know how to advise about social distancing to minimize the risks of community exposure. |  |  |  |  |  |  |
| 14. I am familiar with the local emergency response system for coronavirus |  |  |  |  |  |  |
| 15. I would be considered a key leadership character in my community in coronavirus outbreak. |  |  |  |  |  |  |
| 16. I consider myself prepared for the management of coronavirus virus. |  |  |  |  |  |  |
| *Response* | | | | | | |
| 1. I am familiar with the scope of my role in coronavirus as a healthcare provider. |  |  |  |  |  |  |
| 2. I am reasonably confident in my abilities in coronavirus as a member of a healthcare team. |  |  |  |  |  |  |
| 3. I would feel confident in my abilities as a direct care provider or first responder in coronavirus. |  |  |  |  |  |  |
| 4. I can care for coronavirus patients independently without any supervision. |  |  |  |  |  |  |
| 5. I can manage the common symptoms and reactions of coronavirus |  |  |  |  |  |  |
| 6. I would feel confident implementing social distancing plans, infection control plans, hygiene literacy training, and  similar functions. |  |  |  |  |  |  |
| 7. I can identify possible indicators of mass exposure evidenced by a clustering of patients with similar symptoms. |  |  |  |  |  |  |
| 8. As a healthcare provider, I would feel confident as a manager or coordinator of a community exposed to coronavirus. |  |  |  |  |  |  |
| 9. I am ready to participate in peer evaluation of skills and governance planning on coronavirus. |  |  |  |  |  |  |
| 10. I am familiar with how to perform focused health assessment for coronavirus. |  |  |  |  |  |  |
| 11. I am accepted as a legitimate authority for coronavirus awareness/ prevention in the community |  |  |  |  |  |  |
| **Community Health Worker Employer Survey** | | | | | |  |
| 1. I recommend my profession as a good place to work |  |  |  |  |  |  |
| 2. My co-workers & I have a good working relationship |  |  |  |  |  |  |
| 3. My supervisor/team leader treats me with respect |  |  |  |  |  |  |
| 4. My supervisor/team leader provides me with opportunities to demonstrate my leadership skills |  |  |  |  |  |  |
| 5. My workload is reasonable |  |  |  |  |  |  |
| 6. My supervisor supports my need to balance work and family issues |  |  |  |  |  |  |
| 7. Employees learn from one another as they do their work |  |  |  |  |  |  |
| 8. Supervisors/team leaders in my work unit support employee development |  |  |  |  |  |  |
| 9. Supervisors/team leaders work well with employees of different backgrounds |  |  |  |  |  |  |
| 10. Communication between senior leadership and employees is good in my organization |  |  |  |  |  |  |
| 11. Creativity and innovation are rewarded |  |  |  |  |  |  |
| 12. My pay and employment benefits are reasonable |  |  |  |  |  |  |
| 13. My job security and contract is reasonable |  |  |  |  |  |  |
| **Self-reported Performance of MCH Workers- Nepal** | | | | | |  |
| *Please answer with respect to the last 3 months, since coronavirus pandemic* | | | | | |  |
| 1. I am satisfied with delivery of services for ANC services |  |  |  |  |  |  |
| 2. I am satisfied with delivery of services for referrals for ANC |  |  |  |  |  |  |
| 3. I am satisfied with delivery of services for births attended |  |  |  |  |  |  |
| 4. I am satisfied with delivery of services for prior referrals for birth care |  |  |  |  |  |  |
| 5. I am satisfied with delivery of services for postnatal care |  |  |  |  |  |  |
| 6. I am satisfied with delivery of services for birth complications managed and/or referred |  |  |  |  |  |  |
| 7. I am satisfied with delivery of services for newborn care |  |  |  |  |  |  |
| 8. I am satisfied with delivery of services for newborn complications managed and/or referred |  |  |  |  |  |  |
| 9. I am satisfied with delivery of services for Children screened |  |  |  |  |  |  |
| 10. I am satisfied with delivery of services for Family planning |  |  |  |  |  |  |
| 11. I am satisfied with delivery of services for Emergency tasks performed |  |  |  |  |  |  |
| 12. I am satisfied with delivery of services for Emergency calls handled |  |  |  |  |  |  |
