# Supplementary material for: Infection preparedness of community health workers: implications for maternal and neonatal health services in Pakistan
Source: Prim Health Care Res Dev. 2022 May 2;23:e27. doi: 10.1017/S1463423622000081 (PMC9112673; doi:10.1017/S1463423622000081)

**Appendix B:**

**Map of Punjab indicating districts where data collection took place for this study**


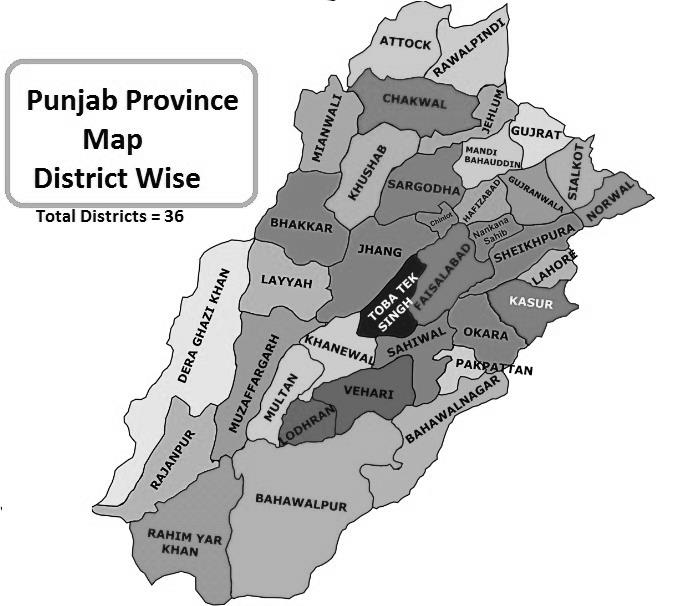


Public domain map of the province of Punjab, not under copyright, retrieved from the website:

<https://www.politicpk.com/punjab-province-list-tehsils-districts-divisions-%D8%B5%D9%88%D8%A8%DB%81-%D9%BE%D9%86%D8%AC%D8%A7%D8%A8/>

| Table: Data collection across 35 districts of Punjab, divided into North Punjab (23 districts) and South Punjab (12 districts). | | | |
| --- | --- | --- | --- |
| **No** | **District** | **Frequency** | **Percentage** |
| **North Punj**a**b** | | | |
| 1 | Attock | 12 | 3.4 |
| 2 | Chakwal | 12 | 3.4 |
| 3 | Chiniot | 9 | 2.6 |
| 4 | Hafizabad | 10 | 2.9 |
| 5 | Faisalabad | 7 | 2.0 |
| 6 | Gujranwala | 2 | 0.6 |
| 7 | Lahore | 10 | 2.9 |
| 8 | Jhang | 5 | 1.4 |
| 9 | Kasur | 7 | 2.0 |
| 10 | Jehlum | 1 | 0.3 |
| 11 | Khushab | 10 | 2.9 |
| 12 | Mandi Bahauddin | 24 | 6.9 |
| 13 | Mianwali | 24 | 6.9 |
| 14 | Narowal | 12 | 3.4 |
| 15 | Nankana Sahab | 30 | 8.6 |
| 16 | Okara | 4 | 1.1 |
| 17 | Pakpattan | 6 | 1.7 |
| 18 | Rawalpindi | 8 | 2.3 |
| 19 | Sargodha | 17 | 4.9 |
| 20 | Sheikupura | 4 | 1.1 |
| 21 | Sialkot | 7 | 2.0 |
| 22 | Toba Tek Singh | 10 | 2.9 |
| 23 | Sahiwal | 18 | 5.1 |
| **South Punjab** | | | |
| 1 | Bahalwalpur | 7 | 2.0 |
| 2 | Bahawalnagar | 13 | 3.7 |
| 3 | Dera Ghazi Khan | 2 | 0.6 |
| 4 | Khanewal | 9 | 2.6 |
| 5 | Layyah | 10 | 2.9 |
| 6 | Lodhran | 7 | 2.0 |
| 7 | Multan | 13 | 3.7 |
| 8 | Muzaffargarh | 9 | 2.6 |
| 9 | Rahim Yar khan | 10 | 2.9 |
| 10 | Rajan Pur | 5 | 1.4 |
| 11 | Vehari | 10 | 2.9 |
| 12 | Bhakkar | 6 | 1.7 |
|  | **TOTAL** | **350** | **100%** |


Supplement: Supplementary file 1 [file phcsup.zip › S1463423622000081sup002.docx]
